# Epidemiology of hallux valgus surgery in Italy: A nationwide study from 2001 to 2016

**DOI:** 10.1002/jeo2.70142

**Published:** 2025-02-13

**Authors:** Umile Giuseppe Longo, Rocco Papalia, Alessandro Mazzola, Sergio De Salvatore, Andrea Marinozzi, Stefano Campi, Ilaria Piergentili, Margaux D'Hooghe, Stefano Zaffagnini, Kristian Samuelsson, Vincenzo Denaro

**Affiliations:** ^1^ Fondazione Policlinico Universitario Campus Bio‐Medico Roma Italy; ^2^ Research Unit of Orthopaedic and Trauma Surgery, Department of Medicine and Surgery Università Campus Bio‐Medico di Roma Roma Italy; ^3^ IRCCS Ospedale Pediatrico Bambino Gesù Rome Italy; ^4^ CNR‐IASI, Laboratorio di Biomatematica, Consiglio Nazionale delle Ricerche Istituto di Analisi dei Sistemi ed Informatica Rome Italy; ^5^ Department of Medicine University of Navarra Pamplona Spain; ^6^ Clinica Ortopedica e Traumatologica II IRCCS Istituto Ortopedico Rizzoli Bologna Italy; ^7^ Sahlgrenska Sports Medicine Center Gothenburg Sweden; ^8^ Department of Orthopaedics Institute of Clinical Sciences, Sahlgrenska Academy University of Gothenburg Gothenburg Sweden; ^9^ Department of Orthopaedics Sahlgrenska University Hospital Mölndal Sweden

**Keywords:** epidemiology, hallux, Italy, surgery, valgus

## Abstract

**Purpose:**

This study intended to estimate the annual number of hallux valgus surgical procedures in Italy and the patients' epidemiological features. A secondary goal was to compare the demographic differences in access to hallux valgus surgery amongst three Italian macroregions.

**Methods:**

The analysis was conducted by using the National Hospital Discharge Records database provided by the Italian Ministry of Health.

**Results:**

721,514 surgical procedures for Acquired Hallux valgus were performed. The cumulative incidence was 88.2 procedures for every 100,000 Italian residents. The highest number of procedures was found in the 60–64 age class. 91.2% of patients were females. The mean length of hospitalisation was 2.1 ± 2.2 days. Patients aged 95–99 had more days of hospitalisation on average. 51.9% of procedures were performed in the North, 25.7% in the Centre and 22.4% in the South. 98.5% of patients from the North received surgical treatment in the same macroregion of domicile: 90% in the Centre and 78.5% in the South. The main primary procedure was: bunionectomy with soft tissue correction and osteotomy of the first metatarsal (79.9%, 77.51 International Classification of Diseases, Ninth Revision, Clinical Modification code).

**Conclusions:**

The socio‐economic burden of hallux valgus surgery in Italy is relevant. The incidence of hallux valgus surgery has progressively increased between 2001 and 2012 and decreased from 2012 to 2016. A geographically unequal distribution of procedures between the three Italian macroregions was pointed out. Migratory flows of patients from the South to the North for undergoing the procedure were observed.

**Level of Evidence:**

Level III.

**Abbreviations**
ICD‐9‐CM: International Classification of Diseases, Ninth Revision, Clinical Modification
NHS: Italian National Health Service

## INTRODUCTION

Due to the variety of hallux valgus clinical presentations, it is likely that a number of factors contribute to the illness [26]. However, the exact pathogenetic mechanism of hallux valgus development still remains under debate [6]. Morphological and biomechanical factors (including anatomical increase of the metatarsal length, rounded articular surface, metatarsus primus varus, pes planus, first‐ray hypermobility and ligamentous laxity), have been suspected as potential aetiologic factors but there are no enough scientific evidence to support or refute their roles in the pathogenesis of the disease [6, 13, 15, 20, 26]. Hallux valgus is considered the most common forefoot problem in the adult population [19]. According to estimates, one third of the population in western countries suffers from hallux valgus deformity [4]. Despite the fact that the peak onset happens between thirty and sixty years of age, the first changes are more likely to happen during adolescence or earlier for juvenile hallux valgus [3]. The male/female ratio of patients requiring surgical correction is 1/15 [27, 29]. The higher prevalence in women is believed to be caused by the use of high‐heeled shoes with a narrow toe box [9]. Accordingly, ballet dancers have shown higher rates of disease than the general population [7, 19]. To date, only Finland's national incidence rates of hallux valgus have been documented [25]. There are not enough studies on the epidemiology of patients with hallux valgus deformity and their treatment options in the Italian population. To better address the future of this surgery and the associated health service planning, it is crucial to investigate the demographic trends of patients with hallux valgus deformity receiving surgery. Equity in access to healthcare is one of the basic concepts of the Italian National Health Service (NHS): patients in Italy get universal access to the NHS for free. Based on official data sources such as hospitalisation records, this study intended to estimate, from 2001 to 2016, the annual number of hallux valgus surgical procedures performed in Italy and the demographic features of patients. A secondary goal was to investigate demographic differences in access to hallux valgus surgery amongst the three Italian macroregions (North, Centre and South).

## MATERIALS AND METHODS

The analysis was conducted by using the National Hospital Discharge Records database provided by the Italian Ministry of Health. All methods were performed in accordance with the relevant guidelines and regulations. Data included in the present study refers to the period from 2001 to 2016. This official database contains data about all national hospitalisations occurring in the Italian public and private care settings. In these records are included the patient's age, sex, region of domicile, region of hospitalisation, length of the hospitalisation (in days), diagnoses and procedures. Diagnoses and procedures are coded by the International Classification of Diseases, Ninth Revision, Clinical Modification (ICD‐9‐CM). The Acquired Hallux valgus diagnosis was defined by the 733.0 ICD‐9‐CM code. All analyses refer to the Italian adult population (patients over 15 years of age). Data relative to the Italian adult population were obtained from the National Institute for Statistics for each year. Italy was divided into three macroregions: the North, the Centre and the South. The North includes the regions of Liguria, Lombardy, Piedmont, Aosta Valley, Emilia–Romagna, Friuli–Venezia Giulia, Trentino—South Tyrol and Veneto. The Centre includes the regions of Lazio, Marche, Tuscany and Umbria. The South includes the regions of Abruzzo, Basilicata, Calabria, Campania, Molise, Apulia, Sardinia and Sicily.

### Statistics

Descriptive statistical analyses were performed. Frequencies and percentages for categorical variables and means and standard deviations for continuous variables were calculated. Incidence rates were calculated as the number of cases divided by the amount of the Italian population over 15 years of age per 100,000 (cases/population*100,000). The Statistical Package for Social Sciences IBM SPSS Statistics for Windows, Version 26.0. Armonk, NY: IBM Corp and Microsoft Excel (2019) were used to perform all data analysis.

## RESULTS

### Demographics

**Figure 1:**
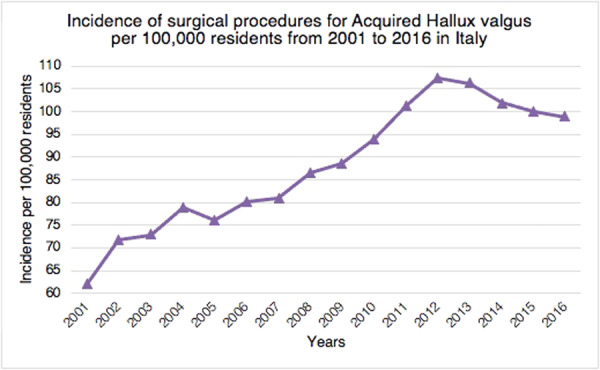
Incidence of surgical procedures for acquired hallux valgus (+15 years of age) per 100,000 residents from 2001 to 2016 in Italy.

Overall, between 2001 and 2016, 721,514 procedures for Acquired Hallux valgus were performed in Italy, with a cumulative incidence of 88.2 procedures for every 100,000 Italian residents over 15 years of age. From 2001 to 2016, the incidence of surgical procedures performed for Acquired Hallux valgus increased from 62.1 per 100,000 in 2001 to 98.9 per 100,000 in 2016 (Figure 1).

**Figure 2:**
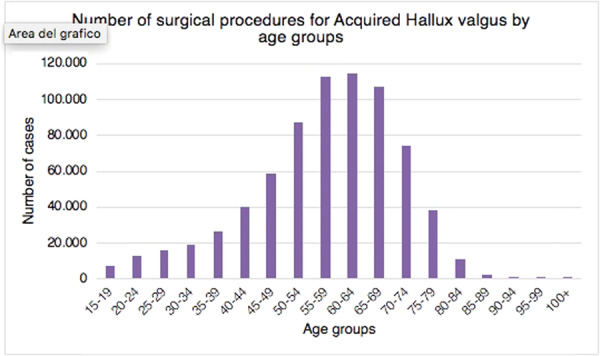
Number of surgical procedures for acquired hallux valgus performed in Italy from 2001 to 2016, stratified for class of age.

Stratifying the analysis by age groups, the highest prevalence of procedures was found in the 55–59 and 60–64 age classes (Figure 2).

The majority of patients who underwent surgical treatment for acquired hallux valgus were females (91.2% of females and 8.8% of males).

**Figure 3:**
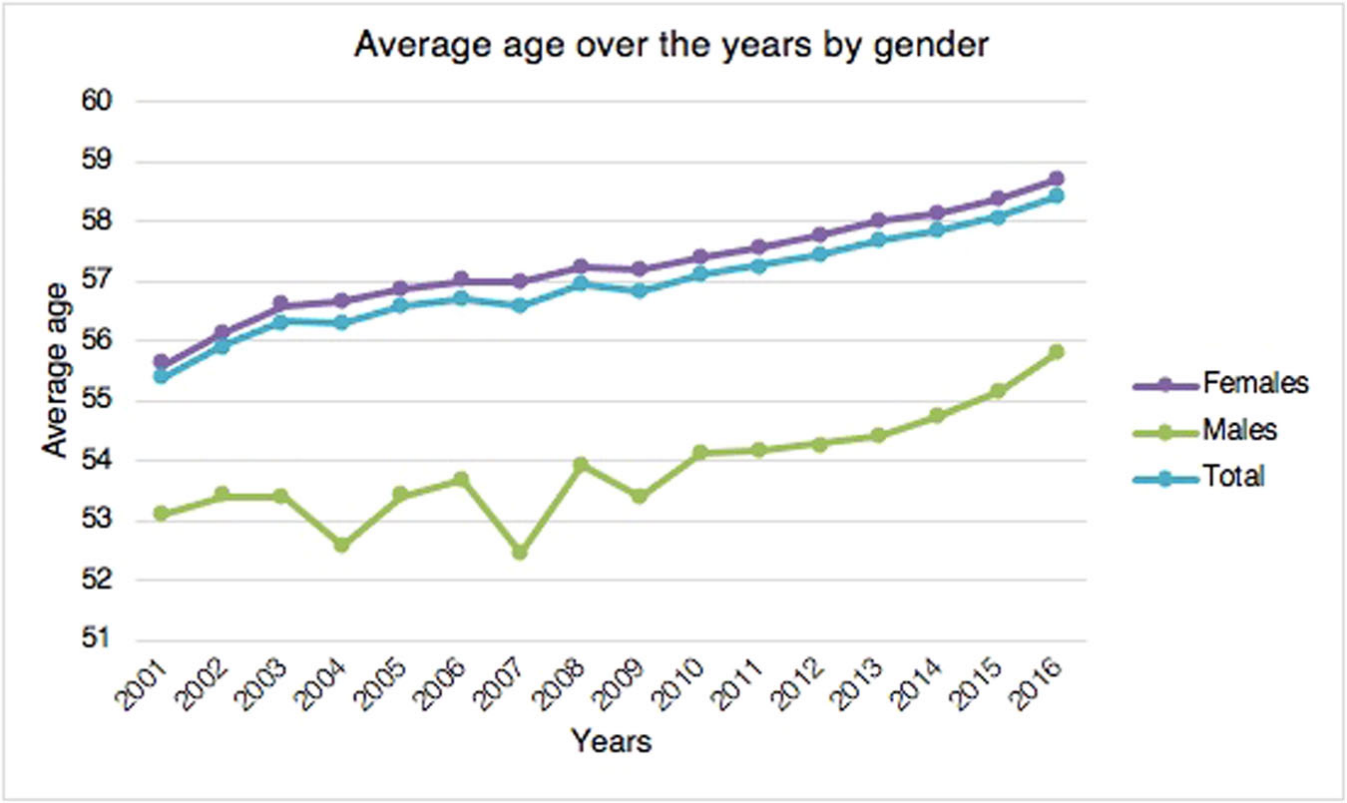
Average age of patients requiring surgery for acquired hallux valgus over the years by gender.

The mean age of patients was 57.1 ± 13.5, with an increasing trend (Figure 3). During the study period, the average age of females was always higher than that of males (Figure 3). The average age of females was 57.4 ± 13.2 years, while the average age of males was 54 ± 16.2 years.

### Length of the hospitalisation

**Figure 4:**
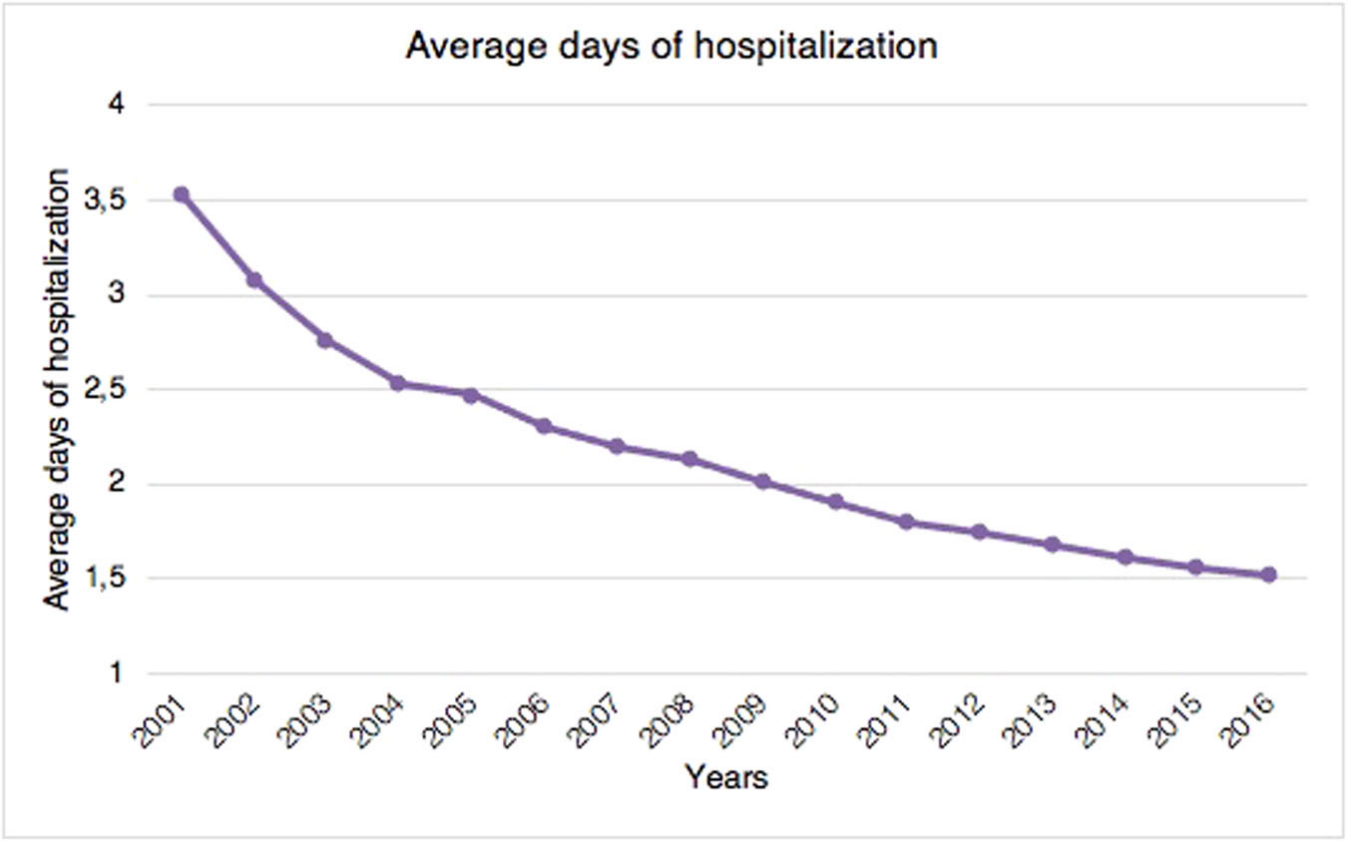
Average days of hospitalisation in the study period.

The mean length of hospitalisation was 2.1 ± 2.2 days. From 2001 to 2016, a decreasing trend of average days of hospital stay was observed (Figure 4).

**Figure 5:**
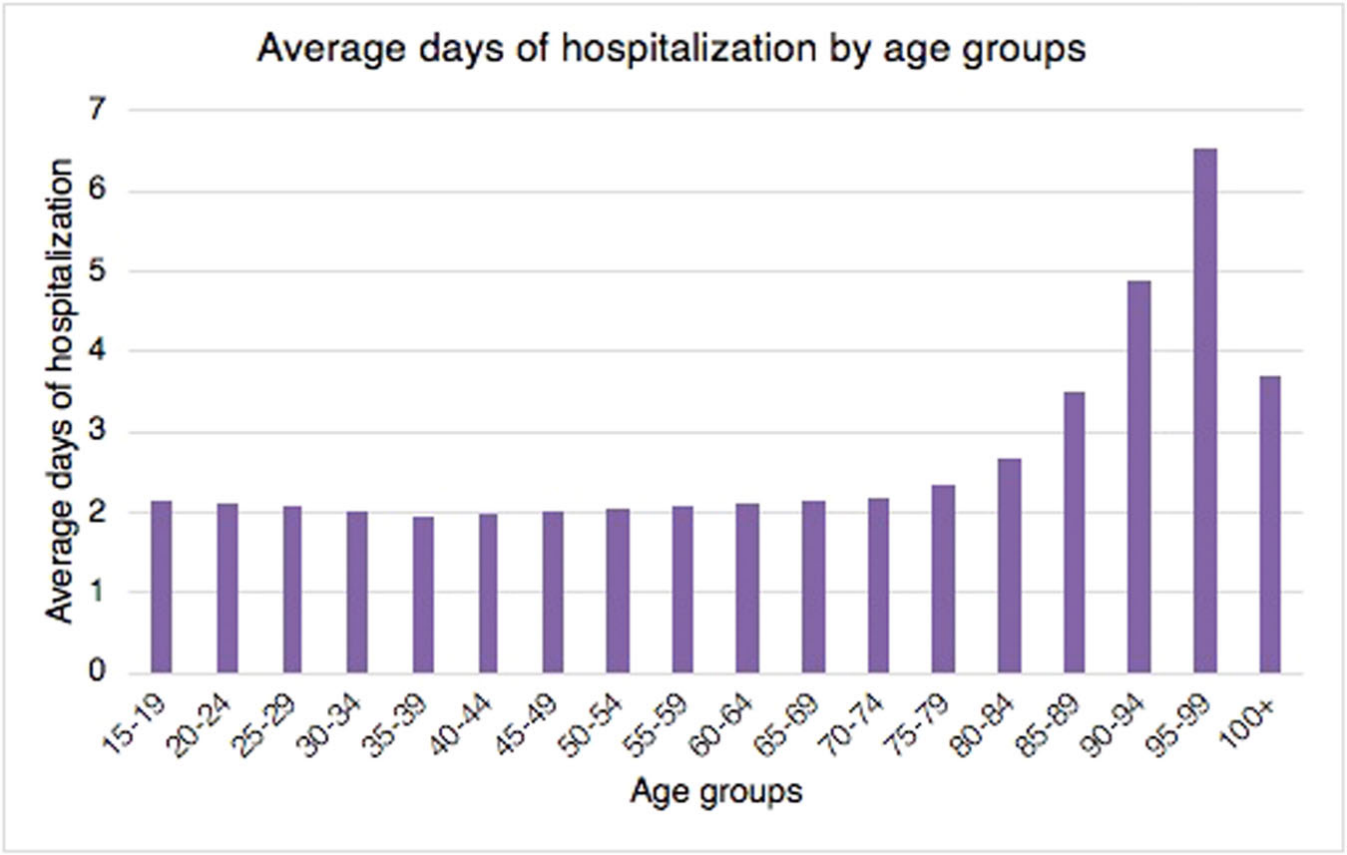
Average days of hospitalisation by age groups in the study period.

Patients aged 95–99 showed more days of hospitalisation on average (Figure 5).

**Figure 6:**
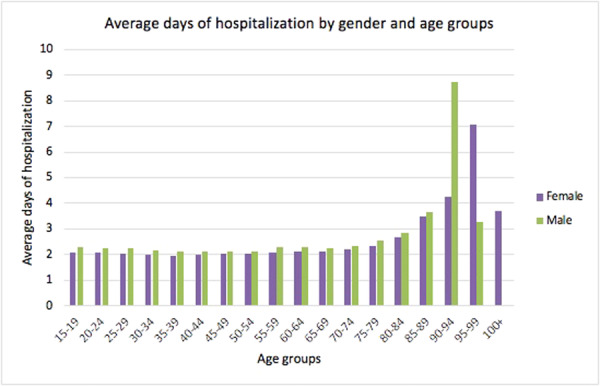
Average days of hospitalisation stratified by gender and age groups in the study period.

Stratified by gender, males between 90 and 94 years of age and women between 95 and 99 years of age showed longer hospital stays (Figure 6).

### Region of hospitalisation and domicile of the patients

**Figure 7:**
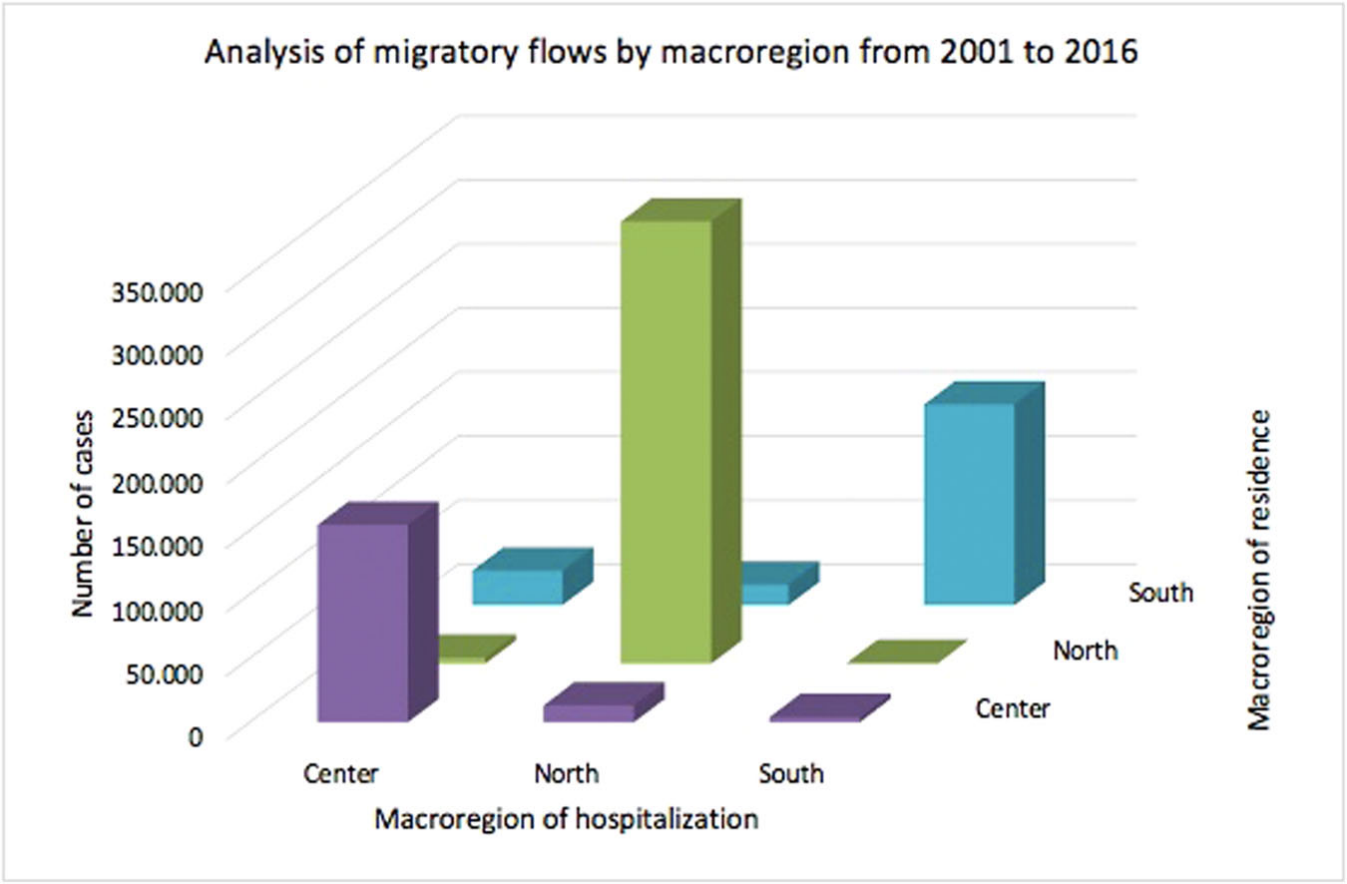
Analysis of migratory flows by macroregion (North, Centre and South) from 2001 to 2016.

During the 16‐year study period, 374,594 procedures for acquired hallux valgus were identified in the North (51.9%), 185,741 cases (25.7%) in the Centre and 161,179 cases (22.4%) in the South. Data about patients' domicile were not available for 649 cases. Therefore, a total of 720,865 procedures for Acquired Hallux valgus with data on patients' domicile were available. 350,306 patients (48.6%) from the North underwent Acquired Hallux valgus surgical treatment from 2009 to 2015, 171,343 patients (23.8%) from the Centre and 199,216 patients (27.6%) from the South (Figure 7).

98.5% of residents of the North received surgical treatment in the same macroregion of domicile. The lowest rates were shown in the Centre (90%) and the South (78.5%).

### Main primary procedures

Between 2001 and 2016, the main primary procedures performed were: bunionectomy with soft tissue correction and osteotomy of the first metatarsal (79.9%, 77.51 ICD‐9‐CM code), other division of bone, tarsals and metatarsals (2.3%, 77.38 ICD‐9‐CM code), other bunionectomy (1.7%, 77.59 ICD‐9‐CM code), other partial ostectomy, tarsals and metatarsals (1.4%, 77.88 ICD‐9‐CM code) and wedge osteotomy, tarsals and metatarsals (1%, 77.28 ICD‐9‐CM code).

## DISCUSSION

The most important finding of this study is the incidence of hallux valgus surgery in the Italian population and its trend over the years. In the current literature, only Finland [25] and Sweden [30] have analysed nationwide trends in primary hallux valgus surgery. Hallux valgus is the most prevalent forefoot issue in adults [2, 10, 19]. Over the study period, the rate of hallux valgus surgical procedures in Italian hospitals increased, thus suggesting an increase in the socio‐economic cost of hallux valgus surgery. The highest prevalence of procedures was found in the 55–59 and 60–64 age classes, thus having a significant impact on the working population. Only a few studies have compared the incidence of hallux valgus surgery across age groups. Data from the Finnish nationwide registry showed that the highest incidence of deformity correction was found in the 50–59 and 60–69 age groups [25]. This is in line with the results of the present study showing the highest prevalence of procedures in Italy in the 55–59 and 60–64 age classes. Geographical differences in hallux valgus surgery rates were seen among Italian regions during the course of the study period. Hallux valgus treatment was mainly carried out in the northern Italian regions. Overall, 51.9% of procedures for acquired hallux valgus were identified in the North, 25.7% in the Centre and 22.4% in the South. It remains unknown whether these variations are due to actual geographic variance in surgical need or patient consent. Saro et al. showed that, in Sweden, forefoot surgery is more common in urban than in rural areas [30]. They argued that it might be due to the greater availability of foot‐skilled surgeons in urban areas [30]. Another reason could be that people in rural areas prefer comfortable and larger shoes [30]. They also found that this type of surgery was mainly performed in private clinics rather than in community hospitals [30]. However, the present study lacks statistics regarding the prevalence, incidence, and severity of hallux valgus deformity in the Italian population. For this reason, it is impossible to determine if these geographic differences represent variances in need or clinical practice. 98.5% of residents of the North received surgical treatment in the same macroregion of domicile. The lowest rates were shown in the Centre (90%) and the South (78.5%). These migratory flows throughout Italian areas highlight a topic that needs more research, especially given the traditional will of the government and the Italian Ministry of Health on eliminating health inequities. Results of the present study showed that gender distribution (8.8% men and 91.2% women) is consistent with the current literature [23, 25, 30]. The average age of females was always higher than males. Men and women are thought to have different etiologies for hallux valgus [23]. High‐heeled shoes with a narrow toe box usage are considered a risk factor for developing deformity in women [5, 22], whereas pes planus and higher body mass index are risk factors in men [23]. Previous studies have confirmed that painful hallux valgus can be effectively treated with surgical correction [28, 31, 32, 34]. In contrast, orthoses just offer temporary symptom alleviation [31, 32]. Our results showed a progressive increase in the incidence of surgical procedures for hallux valgus in Italy from 2001 to 2012. This is in line with the superiority of surgical correction versus conservative management argued in the literature. From 2012 to 2016, a slight decrease in surgical correction incidence began. It could be due to a restriction of indications for surgery in patients with the deformity and the improvement of conservative methods. However, no studies are available to confirm this hypothesis.

In Italy, the hospitalisation length of stay following hallux valgus correction significantly decreased from 2001 to 2016. This trend may be linked to a general rearrangement of the length of stay for economic reasons by hospitals. It may also be related to the improvement of minimally invasive surgical techniques and faster recovery after anaesthetic procedures over the years. However, no data may confirm this claim.

When nonoperative measures fail, surgery is necessary, however the results are not always predictable. Unfavourable outcomes include infection, hallux varus residual deformity and recurrence [1, 12, 21, 33]. Literature showed that problems following hallux valgus surgery are frequent; hence, surgical therapy should be carefully considered, especially in the case of treating asymptomatic or minor abnormalities [1, 12]. By far, the main primary procedure performed from 2001 to 2016 in Italy was bunionectomy with soft tissue correction and osteotomy of the first metatarsal (79.9%, 77.51 ICD‐9‐CM code). Many surgical osteotomies of the first metatarsophalangeal joint have been described [17, 18, 24], with no techniques showing superiority among others [8, 11, 14, 16].

The present study's strengths include complete national coverage, a significant study period, the use of official national data, and the inclusion of the whole adult population of Italy. All hospitals, whether public or private, are required to gather data for the Italian Ministry of Health.

Limitations of the present study are: first, the ICD‐9‐CM, which is the basis for all reported diagnoses and procedures, is based on administrative data from various hospitals and areas. Due to the numerous hospitals involved, it is difficult to discover diagnoses or coding inaccuracies. Second, the ICD‐9‐CM allows for the use of several codes for the same surgical procedure. Our investigation revealed significant regional variations. It is unclear from our data whether this variability is linked to patients' and attitudes or actual geographic variance in surgical need. Moreover, the generic and anonymized fashion of the ICD9‐CM codes resulted in the impossibility of determining differences in terms of hospitalisation length on the basis of the surgical technique performed or between public/private hospitals.

## CONCLUSIONS

In conclusion, these data support the increased socio‐economic burden of hallux valgus surgery in Italy, which has a significant impact on the working population. In Italy, the overall incidence of hallux valgus surgery has significantly increased between 2001 and 2012. In contrast, a slight decrease from 2012 to 2016 has been observed. In line with the literature, women are markedly more affected than men in the sixth and seventh decades of life. The most popular surgical procedure for treating hallux valgus is the first metatarsal osteotomy. Hospitalisation length stay significantly decreased over the study period. Our study showed the geographically unequal distribution of procedures between the three Italian macroregions: in the northern regions, hallux valgus treatment was mainly carried out. Moreover, macroregional migratory flows from the South to the North have been reported. Further investigations are needed in order to shed light on regional public health system inequalities in Italy.

## AUTHOR CONTRIBUTIONS

*Manuscript preparation, study design, database interpretation and manuscript revision*: Umile Giuseppe Longo, Alessandro Mazzola, Rocco Papalia and Vincenzo Denaro. *Manuscript preparation, database interpretation and statistical analysis*: Umile Giuseppe Longo, Alessandro Mazzola and Stefano Zaffagnini. *Manuscript preparation, figures and tables preparation and study design*: Alessandro Mazzola, Ilaria Piergentili, Sergio De Salvatore and Kristian Samuelsson. *Manuscript preparation and database interpretation*: Umile Giuseppe Longo, Alessandro Mazzola and Stefano Campi. *Study design and manuscript revision*: Umile Giuseppe Longo, Alessandro Mazzola and Vincenzo Denaro. The authors read and approved the final manuscript.

## CONFLICT OF INTEREST STATEMENT

The authors declare no conflicts of interest.

## ETHICS STATEMENT

The Institutional Review Board of Campus Bio‐Medico University of Rome ruled that no formal ethics approval was required in this particular case and the need to obtain informed consent was waived based on the retrospective design and anonymization of patient identifiers (Prot. number: 113/20 (OSS) ComEt UCBM). All methods were performed in accordance with the relevant guidelines and regulations. All data were obtained by the Direzione Generale della Programmazione Sanitaria—Banca Dati SDO of the Italian Ministry of Health.

## Data Availability

The data sets used and/or analysed during the current study are available from the corresponding author on reasonable request. The access to the database is on request. All data were obtained by the Direzione Generale della Programmazione Sanitaria—Banca Dati SDO of the Italian Ministry of Health.

## References

[1] Akhtar, S. , Malek, S. & Hariharan, K. (2016) Hallux varus following scarf osteotomy. The Foot, 29, 1–5. Available from: 10.1016/j.foot.2016.09.004 27888785

[2] Black, J. & Hale, W. (1987) Prevalence of foot complaints in the elderly. Journal of the American Podiatric Medical Association, 77, 308–311. Available from: 10.7547/87507315-77-6-308 3612508

[3] Coughlin, M.J. (1995) Juvenile hallux valgus: etiology and treatment. Foot & Ankle International, 16, 682–697. Available from: 10.1177/107110079501601104 8589807

[4] Coughlin, M.J. (1996) Instructional course lectures, The American Academy of Orthopaedic Surgeons—hallux valgus*†. The Journal of Bone & Joint Surgery, 78, 932–966. Available from: 10.2106/00004623-199606000-00018 8666613

[5] Coughlin, M.J. & Thompson, F.M. (1995) The high price of high‐fashion footwear. Instructional Course Lectures, 44, 371–377. 7797875

[6] Doty, J.F. & Harris, W.T. (2018) Hallux valgus deformity and treatment. Foot and Ankle Clinics, 23, 271–280. Available from: 10.1016/j.fcl.2018.01.007 29729801

[7] Einarsdottir, H. , Troell, S. & Wykman, A. (1995) Hallux valgus in ballet dancers: a myth? Foot & Ankle International, 16, 92–94. Available from: 10.1177/107110079501600208 7767454

[8] Ferrari, J. , Higgins, J.P. & Prior, T.D. (2004) Interventions for treating hallux valgus (abductovalgus) and bunions. Cochrane Database of Systematic Reviews, 1, CD000964. 10.1002/14651858.CD000964.pub2 14973960

[9] Frey, C. , Thompson, F. , Smith, J. , Sanders, M. & Horstman, H. (1993) American Orthopaedic Foot and Ankle Society women's shoe survey. Foot & Ankle, 14, 78–81. Available from: 10.1177/107110079301400204 8454237

[10] Gould, N. , Schneider, W. & Ashikaga, T. (1980) Epidemiological survey of foot problems in the continental United States: 1978‐1979. Foot & Ankle, 1, 8–10. Available from: 10.1177/107110078000100104 6115797

[11] van Groningen, B. , van der Steen, M.C. , Reijman, M. , Bos, J. & Hendriks, J.G.E. (2016) Outcomes in chevron osteotomy for hallux valgus in a large cohort. The Foot, 29, 18–24. Available from: 10.1016/j.foot.2016.09.002 27888787

[12] Hecht, P.J. & Lin, T.J. (2014) Hallux valgus. Medical Clinics of North America, 98, 227–232. Available from: 10.1016/j.mcna.2013.10.007 24559871

[13] Inman, V.T. (1974) Hallux valgus: a review of etiologic factors. Orthopedic Clinics of North America, 5, 59–66. Available from: 10.1016/S0030-5898(20)31240-2 4809546

[14] Klugarova, J. , Hood, V. , Bath‐Hextall, F. , Klugar, M. , Mareckova, J. & Kelnarova, Z. (2017) Effectiveness of surgery for adults with hallux valgus deformity: a systematic review. JBI Database of Systematic Reviews and Implementation Reports, 15, 1671–1710. Available from: 10.11124/JBISRIR-2017-003422 28628523

[15] Longo, U.G. , Marinozzi, A. , Petrillo, S. , Spiezia, F. , Maffulli, N. & Denaro, V. (2013) Prevalence of accessory ossicles and sesamoid bones in hallux valgus. Journal of the American Podiatric Medical Association, 103, 208–212. Available from: 10.7547/1030208 23697726

[16] Maffulli, N. , Longo, U.G. , Marinozzi, A. & Denaro, V. (2011) Hallux valgus: effectiveness and safety of minimally invasive surgery. A systematic review. British Medical Bulletin, 97, 149–167. Available from: 10.1093/bmb/ldq027 20710024

[17] Maffulli, N. , Longo, U.G. , Oliva, F. , Denaro, V. & Coppola, C. (2009) Bosch osteotomy and scarf osteotomy for hallux valgus correction. Orthopedic Clinics of North America, 40, 515–524. Available from: 10.1016/j.ocl.2009.06.003xi‐x 19773057

[18] Mann, R.A. (1990) Decision‐making in bunion surgery. Instructional Course Lectures, 39, 3–13. 2186117

[19] Mann, R.A. & Coughlin, M.J. (1981) Hallux valgus—etiology, anatomy, treatment and surgical considerations. Clinical Orthopaedics and Related Research, 157, 31–41. Available from: 10.1097/00003086-198106000-00008 7249460

[20] Marinozzi, A. , Longo, U.G. , Cazzato, L. , Martinelli, N. , Maffulli, N. & Denaro, V. (2011) Bilateral tibial hallux sesamoid agenesis and fibular hallux sesamoid hypoplasia in a patient with bilateral hallux valgus. Journal of the American Podiatric Medical Association, 101, 452–455. Available from: 10.7547/1010452 21957278

[21] Martinelli, N. , Cancilleri, F. , Marineo, G. , Marinozzi, A. , Longo, U.G. & Denaro, V. (2012) Pseudarthrosis after percutaneous distal osteotomy in hallux valgus surgery: a case report. Journal of the American Podiatric Medical Association, 102, 78–82. Available from: 10.7547/1020078 22232327

[22] Menz, H.B. & Morris, M.E. (2005) Footwear characteristics and foot problems in older people. Gerontology, 51, 346–351. Available from: 10.1159/000086373 16110238

[23] Nguyen, U.S.D.T. , Hillstrom, H.J. , Li, W. , Dufour, A.B. , Kiel, D.P. , Procter‐Gray, E. et al. (2010) Factors associated with hallux valgus in a population‐based study of older women and men: the MOBILIZE Boston Study. Osteoarthritis and Cartilage, 18, 41–46. Available from: 10.1016/j.joca.2009.07.008 19747997 PMC2818204

[24] Oliva, F. , Longo, U.G. & Maffulli, N. (2009) Minimally invasive hallux valgus correction. Orthopedic Clinics of North America, 40, 525–530. Available from: 10.1016/j.ocl.2009.06.005x 19773058

[25] Partio, N. , Mäenpää, H. , Huttunen, T. , Haapasalo, H. , Laine, H.J. & Mattila, V.M. (2019) Incidence of hallux valgus primary surgical treatment. Finnish nationwide data from 1997 to 2014. Foot and Ankle Surgery, 25, 761–765. Available from: 10.1016/j.fas.2018.10.001 31796164

[26] Perera, A.M. , Mason, L. & Stephens, M.M. (2011) The pathogenesis of hallux valgus. Journal of Bone and Joint Surgery, 93, 1650–1661. Available from: 10.2106/JBJS.H.01630 21915581

[27] Piqué‐Vidal, C. , Solé, M.T. & Antich, J. (2007) Hallux valgus inheritance: pedigree research in 350 patients with bunion deformity. The Journal of Foot and Ankle Surgery, 46, 149–154. Available from: 10.1053/j.jfas.2006.10.011 17466240

[28] Robinson, A.H.N. & Limbers, J.P. (2005) Modern concepts in the treatment of hallux valgus. The Journal of Bone and Joint Surgery. British Volume, 87, 1038–1045. Available from: 10.1302/0301-620X.87B8.16467 16049235

[29] Saro, C. , Andren, B. , Wildemyr, Z. & Felländer‐Tsai, L. (2007) Outcome after distal metatarsal osteotomy for hallux valgus: a prospective randomized controlled trial of two methods. Foot & Ankle International, 28, 778–787. Available from: 10.3113/FAI.2007.0778 17666169

[30] Saro, C. , Bengtsson, A.S. , Lindgren, U. , Adami, J. , Blomqvist, P. & Felländer‐Tsai, L. (2008) Surgical treatment of hallux valgus and forefoot deformities in Sweden: a population‐based study. Foot & Ankle International, 29, 298–304. Available from: 10.3113/FAI.2008.0298 18348826

[31] Torkki, M. , Malmivaara, A. , Seitsalo, S. , Hoikka, V. , Laippala, P. & Paavolainen, P. (2001) Surgery vs orthosis vs watchful waiting for hallux valgus: a randomized controlled trial. Journal of the American Medical Association, 285, 2474–2480. Available from: 10.1001/jama.285.19.2474 11368700

[32] Torkki, M. , Malmivaara, A. , Seitsalo, S. , Hoikka, V. , Laippala, P. & Paavolainen, P. (2003) Hallux valgus: immediate operation versus 1 year of waiting with or without orthoses: a randomized controlled trial of 209 patients. Acta Orthopaedica Scandinavica, 74, 209–215. Available from: 10.1080/00016470310013987 12807332

[33] Trnka, H.J. , Zettl, R. , Hungerford, M. , Mühlbauer, M. & Ritschl, P. (1997) Acquired hallux varus and clinical tolerability. Foot & Ankle International, 18, 593–597. Available from: 10.1177/107110079701800913 9310773

[34] Wilson, D.W. (1980) Treatment of hallux valgus and bunions. British Journal of Hospital Medicine, 24, 548–549. 7272540

